# Surgical Treatments for Scapholunate Ligament Injuries and Development of Arthritis in the Wrist: A Systematic Review

**DOI:** 10.1016/j.jhsg.2025.100827

**Published:** 2025-09-13

**Authors:** Eiden Lami, Jack Kramer, Majd Mzeihem, Farid Amirouche

**Affiliations:** ∗ Department of Orthopaedic Surgery, University of Illinois at Chicago, Chicago, IL; † Department of Orthopaedic Surgery, Northshore University Health System, University of Chicago Pritzker School of Medicine, Skokie, IL

**Keywords:** Arthritis, Ligament, Scapholunate, Surgery, Wrist

## Abstract

**Purpose:**

The scapholunate ligament (SLL) is the most injured carpal ligament; however, surgeons do not agree on the best management. Several studies have reported on the after surgery functional outcomes of different surgical techniques, with varying results. However, there is little literature on the development of arthritis in the context of a SLL injury. The goal of this review was to examine the current literature to investigate the relationship between an SLL injury and the development of arthritis in the wrist and hand.

**Methods:**

We performed a systematic literature review on surgical treatments for SLL injuries. This review included 37 studies that met the inclusion criteria for treatment outcomes and arthritis development. We reported both demographic and radiological results, which included arthritis prevalence and pattern. Methodological quality was assessed using the Downs and Black checklist.

**Results:**

In total, 784 injured wrists were analyzed across the 37 studies, and postoperative arthritis occurrences across treatment types were as follows, capsulodesis (10.6%), tenodesis (13.6%), ligamentoplasty (14.6%), bone-ligament-bone (31.5%), and debridement (5.3%), with scapholunate advanced collapse III being the most common arthritic pattern. The mean age of patients was 39.8 years. The delay between injury and treatment averaged 13.2 months.

**Conclusions:**

Our systematic review highlights the variability in arthritis patterns following SLL injuries and underscores the lack of consensus regarding management strategies. We observed the highest rates of arthritis using the bone-ligament-bone method and the lowest rates using the debridement technique.

**Type of study/level of evidence:**

Therapeutic IIA.

The scapholunate ligament (SLL) stabilizes the scaphoid and lunate, especially during flexion and extension, as the two bones rotate along the triquetrum.^1^ Injuries to the SLL and other carpal ligaments because of trauma typically result from falls and are commonly associated with an extended wrist with an ulnar deviation.^2^ The SLL is the most injured carpal ligament, with approximately five percent of wrist sprains involving a tear of the SLL.^3^

If left untreated, SLL injuries lead to osteoarthritis (OA), but there is no consensus on the best way to manage SLL injuries.^4^ Some chronic SLL injuries develop other complications, such as dorsal intercalated segment instability, and progressively worsening carpal collapse. Scapholunate advanced collapse (SLAC), a severe degenerative change, presents as the most common pattern of arthritis following SLL injury.^3^^,^^5^^,^^6^ The progression of SLAC typically begins at the radioscaphoid joint. The joint between the capitate and lunate is then affected, followed by the scaphotrapeziotrapezoid joints.^7^ The injury then deteriorates to scapholunate dissociation, leading to perilunate instability and pain. The altered positioning of the carpals can lead to diminished load-bearing capabilities of the wrist, leading to carpal collapse.^5^ It often takes 3–12 months after trauma before dynamic instability develops and SLL dissociation is noted radiologically, with the average time from the initial trauma to SLAC development being unknown and variable.^3^

Surgical treatment is often performed to prevent the progression of arthritis in the wrist. Current literature suggests that surgical treatment can still lead to the formation of wrist arthritis, and there needs to be a consensus among hand surgeons on the best surgical management methods for SLL injuries. Capsulodesis involves reattaching the SLL to the surrounding capsule. Tenodesis uses a tendon graft, typically from the palmaris longus or another tendon, to reconstruct the ligament.^8^ Patients with partial tears can undergo arthroscopic SLL debridement and thermal shrinkage rather than open surgery.^9^ This technique is aimed at alleviating symptoms rather than reconstructing stability and involves removing the torn or degenerated portion of the ligament. The SLL injuries that have progressed to SLAC or other patterns of OA require carpal arthrodesis. Patients undergoing carpal arthrodesis may have a fusion of carpals, known as two-, three-, or four-corner arthrodesis.^10^ Bone-ligament-bone techniques involve securing the ligament using anchors or tunnels in the bone, enhancing anatomical fixation, and potentially improving long-term outcomes. Ligamentoplasty uses autografts or allografts to replace the damaged ligament entirely, restoring joint stability.

Although studies have explored one surgical technique to treat SLL injuries or have looked at specific outcomes, more literature is needed on the association between SLL tears and the development of arthritis. This review explores how SLL injury can lead to arthritis by comparing the type of surgical intervention used for SLL repair and the development of arthritis. The objective was to highlight gaps in existing research and emphasize the need for long-term follow-up studies to better understand these relationships.

## Materials and Methods

### Article selection

**Figure 1:**
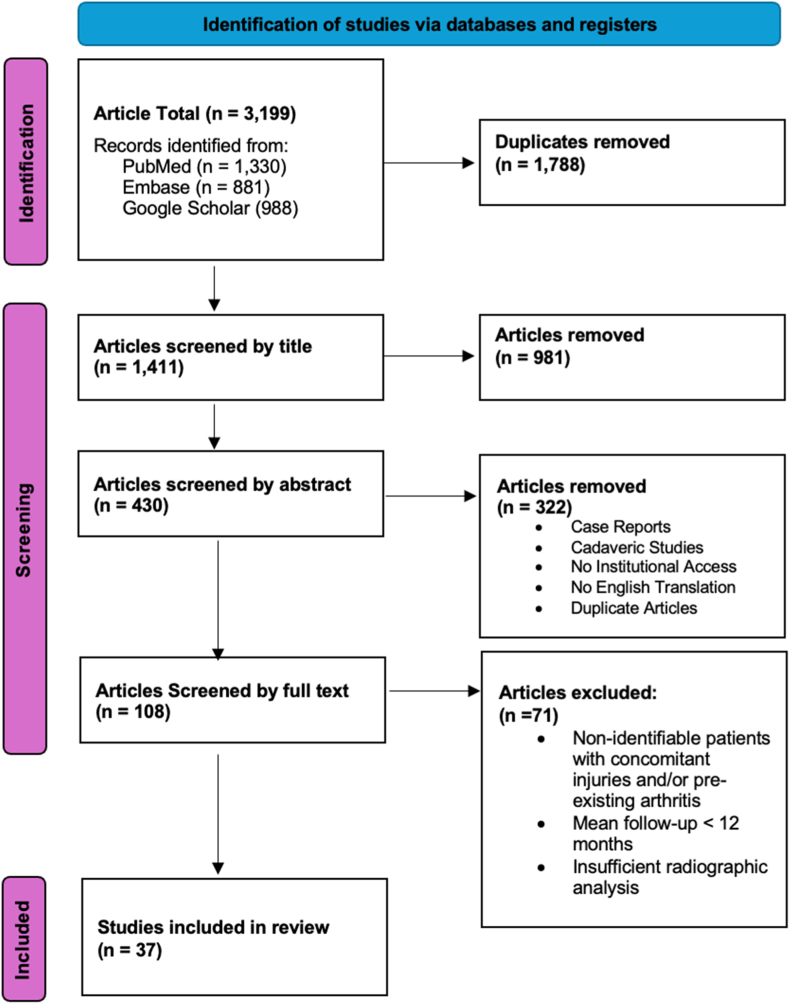
The preferred reporting items for systematic reviews and meta-analyses 2020 flow diagram depiction of articles selection for systematic review based on inclusion criteria.

We used three online databases, PubMed, Embase, and Google Scholar, to search for studies regarding surgical treatment for SLL injuries. The preferred reporting items for systematic reviews and meta-analyses were used in the collection and reporting of data.^11^ The results of all three databases combined 1,411 articles after removing duplicates, which were then screened for relevance. Article titles were used in the initial screening, which resulted in 430 articles. Abstracts were reviewed to further screen for relevance, and 108 articles were yielded. A complete text analysis was used for the final screening round, which yielded 37 articles (Fig. 1).^12^, ^13^, ^14^, ^15^, ^16^, ^17^, ^18^, ^19^, ^20^, ^21^, ^22^, ^23^, ^24^, ^25^, ^26^, ^27^, ^28^, ^29^, ^30^, ^31^, ^32^, ^33^, ^34^, ^35^, ^36^, ^37^, ^38^, ^39^, ^40^, ^41^, ^42^, ^43^, ^44^, ^45^, ^46^ Our inclusion criteria required articles to focus on the surgical treatment of isolated SLL injuries noncomplicated by arthritis. Inclusion required diagnosis to be confirmed with radiography or arthroscopy, and radiography to be used at the last follow-up. The articles included clinical trials, prospective, retrospective studies, and case series with five or more patients. Articles were composed of level III and IV evidence studies.

Excluded articles included cadaveric studies, case reports, non-English articles, articles without institutional access, expert opinions, book chapters, current concepts, abstract-only articles, and reviews. Articles with a mean follow-up time of less than 12 months were excluded. Articles containing patients with concomitant injuries and preexisting arthritis were included if the study used individualized patient data, which could be separated from the isolated SLL injuries without before surgery degenerative changes.

Study quality was assessed using a modified Downs and Black checklist for methodological quality.^8^^,^^9^^,^^47^ The Downs and Black checklist can assess study methodology for randomized and nonrandomized studies. The checklist assesses data reporting, internal and external validity, and power. Studies were scored based on the sum of the checklist, with a maximum score of 28 for randomized studies and 25 for nonrandomized studies. No randomized control trials were included in the systematic review because of a lack of available articles within the inclusion criteria. Comparative studies were included, but no studies were conducted on randomized patient groups.

### Data collection and analysis

The included articles were separated by the surgical method used. Articles were categorized by the procedures: capsulodesis, tenodesis, bone-ligament-bone, ligamentoplasty, and debridement. The terms “arthritis, arthrosis, degeneration, degenerative changes, and SLAC” were counted toward our arthritis results when quantified by the study’s authors. The total count of arthritic wrists within each study was converted into percentages. The pattern of arthritis was recorded when available and used to investigate the significance of arthritic pattern and treatment type, as well as comparison to previous literature such as Watson and Ballet.^7^

Demographic data were collected, such as mean age and range, mean time between injury and intervention, man-woman ratio, and hand dominance. Study data, such as study type, mean follow-up length and range, and number of patients, were also collected. Total, mean, and standard deviation values were calculated for qualifying data categories.

## Results

### Demographic results

Among the included publications were 776 patients representing 784 injured wrists (Table 1). The patient population comprised 465 men, 218 women, and 85 unknowns. The mean number of patients was 18.3, and there were 18.4 wrists within each study group. The mean follow-up time among studies was 56 months. The mean follow-up time was not reported in the two treatment groups of the Wagner et al^46^ study. Complete demographic data are shown in the Table 1, separated by treatment type. The mean age of patients was 39.8 years. One study, Lavernia et al,^23^ did not report the age of patients. The delay between injury and treatment averaged 13.2 months. Six studies did not report delays between treatment and surgery. The mean occurrence of injury to the dominant wrist was 60.6%. Eighteen studies did not report hand dominance.

**Table 1:** Demographic Data

Capsulodesis Studies	Patients (Wrists)	Mean Age and Range	Time to Treatment and Range (Mo)	Mean Follow-Up and Range (Mo)	Gender Ratio (M:F)	Hand Dominance	Downs and Black Score
Shibayama et al,^19^ 2022	5	37 (21–47)	26.2 (2–113)	96 (62–144)	5:0 (100%)	2/5 (40%)	14/25
Chen et al,^20^ 2021	24	51 (18–67)	1.4 (0.1–3)	80 (48–207.5)	16:10 (61.5%)	17/24 (70.8%)	19/25
Moran et al,^8^ 2006	14	41 (17–75)	20 (4–30)	38 (24–75)			18/25
Pomerance,^21^ 2006	17	36 (18–54)	4 (1.5–7)	66 (19–120)	12:5 (70.6%)	12/17 (70.6%)	17/25
Lee et al,^22^ 2023	19	24 (17–27)	2 (1–2.8)	26.5 (24–32)	16:3 (84.2%)	10/19 (52.6%)	19/25
Lavernia et al,^23^ 1992	21	(14–52)	17 (1–84)	33 (18–96)	13:8 (61.9%)	11/21 (52.4%)	16/25
Moran et al,^24^ 2005	31	38 (17–76)	20 (4–84)	54 (24–96)	20:11 (64.5%)		19/25
Szabo et al,^25^ 2002	21 (22)	41 (16–62)		25.2 (12–48)	16:5 (76.2%)	13/22 (59.1%)	18/25
Wintman et al,^26^ 1995	19 (20)	28 (15–55)	17 (3–61)	34 (12–65)	7:13 (35%)	16/20 (80%)	17/25
Carvalho et al,^27^ 2017	14	38 (19–60)		12 (3–17)	9:5 (64.3%)		20/25
Deshmukh et al,^28^ 1999	44	29 (19–46)	54	22 (12–36)	24:20 (54.5%)	28/44 (63.6%)	19/25
van Kampen et al, 2024^15^	14 (16)	45 (8–75)	12 (1–49)	122 (38–237)			21/25
Luchetti et al,^29^ 2010	18	35 (15–57)	10 (2–24)	45 (34–60)	9:9 (50%)	6/18 (33.3%)	19/25
Total	261 (265)	(8–75)	(0.1–113)	(3–237)	147:89	115/190 (60.5%)	
Mean	20.1 (20.4)	37	16.7	50.3	65.4%	58.0%	
SD	9	7.1	14.1	31	15.9%	14.3%	

Tenodesis Studies	Patients (Wrists)	Mean Age and Range	Time to Treatment and Range (Mo)	Mean Follow-Up and Range (Mo)	Gender Ratio (M:F)	Hand Dominance	Downs and Black Score
Nienstedt et al,^40^ 2023	8	44 (29–65)	11 (3–36)	144 (60–168)			18/25
Moran et al,^8^ 2006	15	39	20 (3–24)	36 (24–84)			19/25
Athlani et al,^41^ 2019	20	43 (22–56)		28 (12–49)	15:5 (75%)	9/20 (45%)	20/25
Chabas et al,^42^ 2008	19	43 (23–57)	15 (2–108)	37 (12–60)	16:3 (84.2%)	9/19 (47.4%)	21/25
Links et al,^43^ 2008	21	30 (19–44)	12 (2–23)	29 (24–36)	15:6 (71.4%)	14/21 (66.7%)	22/25
Neinstedt,^44^ 2013	8	40 (28–54)	4 (1.5–12)	165 (144–180)	7:1 (87.5%)		18/25
Pauchard et al,^45^ 2013	20	42.8 (22–56)	13.2 (2–79)	25.1 (12–46)	15:5 (75%)	9/20 (45%)	22/25
van Kampen et al,^15^ 2024	14 (15)	49 (30–66)	12 (2–73)	68.4 (54–139)			21/25
Wagner et al,^46^ 2022∗	12	47.4 (31–71)	9.8 (5–36)	(13–39)	9:0 (100%)		22/25
Wagner et al,^46^ 2022	9	53.5 (40–65)	5.7 (4–8)	(36–120)	8:4 (66.7%)		
Total	146 (147)	(19–71)	(1.5–108)	(12–180)	85:24 (78.0%)	41/80 (51.3%)	
Mean	14.6	43.1	11.4	66.6	80%	51.1%	
SD	3.1	3.8	4.5	52.6	10.5%	8.1%	

Bone-Ligament-Bone Studies	Patients (Wrists)	Mean Age and Range	Time to Treatment and Range (Mo)	Mean Follow-Up and Range (Mo)	Gender Ratio (M:F)	Hand Dominance	Downs and Black Score
Nakamura et al,^12^ 2015	13 (14)	44 (25–75)		38 (24–48)	13:1 (92.9%)		13/25
Dellarosa et al,^13^ 2016	11	38.3 (22–54)	8.8 (5–14)	28.5 (20–38)	8:11 (42.1%)		22/25
Marcuzzi and Leigheb,^14^ 2016	6	41.8 (27–54)		75.7 (46–112)	6:0 (100%)		18/25
van Kampen et al,^15^ 2024	12 (13)	48 (20–78)	14 (1–38)	106.8 (26–143)			21/25
Della Rosa et al,^16^ 2022	31	38 (18–55)	12 (6–34)	68	23:8 (74.2%)	23/31 (74.2%)	20/25
van Kampen et al,^17^ 2015	13	48 (29–66)	16.5 (2–72)	110 (62–136)	12:1 (92.3%)	5/13 (38.5%)	20/25
Gray et al,^18^ 2015	19	46 (22–69)	8 (1.4–24)	98 (35–156)	22:2 (91.7%)		18/25
Total	105 (108)	(18–78)	(2–72)	(20–156)	84:23 (78.5%)	22/44 (63.6%)	
Mean	15 (15.3)	43.4	11.9	75	81.7%	58.8%	
SD	7.4	3.9	3.1	30.1	18.2%	15.0%	

Ligamentoplasty Studies	Patients (Wrists)	Mean Age and Range	Time to Treatment and Range (Mo)	Mean Follow-Up and Range (Mo)	Gender Ratio (M:F)	Hand Dominance	Downs and Black Score
Métairie et al,^35^ 2022	22	39.7 (22–58)	17.9 (3–120)	28 (12–65)	18:4 (81.8%)	15/22 (68.2%)	18/25
Athlani et al,^36^ 2021	19	44.6 (23–61)	12 (5–60)	34 (12–54)	12:7 (63.2%)	13/19 (68.4%)	22/25
Athlani et al,^36^ 2021∗	21	26 (21–34)	10 (3–48)	27 (13–40)	16:5 (76.2%)	16/21 (76.2%)	22/25
Melone et al,^37^ 2012	18	38 (26–49)	1.2 (0.25–4.5)	62 (18–180)		15/18 (83.3%)	13/25
Helfter et al,^38^ 2024	21	40 (22–50)	13.1 (3–48)	14 (6–24)	16:5 (76.2%)	13/21 (61.9%)	17/25
Schweizer and Steiger,^39^ 2022	22	46 (29–68)	6	63 (12–134)	19:3 (86.4%)	13/22 (59.1%)	20/25
Total	123	(21–68)	(0.25–120)	(6–180)	81:24 (77.1%)	85/123 (69.1%)	
Mean	20.5	39.1	10	38	76.8%	69.5%	
SD	1.5	6.5	5.3	18.3	7.8%	8.2%	

Debridement Studies	Patients (Wrists)	Mean Age and Range	Time to Treatment and Range (Mo)	Mean Follow-Up and Range (Mo)	Gender Ratio (M:F)	Hand Dominance	Downs and Black Score
Kim et al,^30^ 2019	41	32 (19–57)	9 (4–14)	68	26:16 (61.9%)		20/25
Fok and Fernandez,^31^ 2015	36	43 (21–63)	12 (6–25)	94.2 (12–120)	20:16 (55.6%)	22/36 (61.1%)	18/25
Darlis et al,^32^ 2006	11	37 (23–50)	7 (4.5–10)	33 (12–76)	8:3 (72.7%)		18/25
Darlis et al,^9^ 2005	16	34 (18–54)	5.4 (3–14)	19 (9–34)	10:6 (62.5%)	9/16 (56.3%)	21/25
Crespo Romero et al,^33^ 2021	20	34.2 (14–53)	36 (18–120)	51 (29–80)	3:17 (15%)	16/20 (80%)	19/25
Ruch and Poehling,^34^ 1996	7	41 (17–59)		34 (24–48)			16/25
Total	131	(14–63)	(3–120)	(9–120)	67:58 (53.6%)	47/72 (65.3%)	
Mean	22	36	13.9	50.0	53.5%	65.8%	
SD	12.8	3.4	11.3	25.3	20.0%	10.2%	

∗ Patients who underwent modified three ligament tenodesis according to Garcia-Elias.

### Radiographic results

One hundred seven wrists developed arthritis across all treatment types, with a 13.6% occurrence after SLL injury. Radiographic results from studies employing the capsulodesis technique for treating SLL injuries reveal varied outcomes regarding the development of arthritis. The most common pattern of arthritis was radiocarpal arthritis and, when specified, was commonly radioscaphoid arthritis. Scapholunate advanced collapse and midcarpal arthritis were also found among the cases. Out of 265 cases reviewed, 28 (10.6%) developed arthritis post-capsulodesis (Table 2).

**Table 2:** Radiographic Results

Capsulodesis Studies	Arthritis	Pattern
Shibayama et al,^19^ 2022	2/5 (40%)	Radioscaphoid
Chen et al,^20^ 2021	4/24 (16.7%)	SLAC
Moran et al,^8^ 2006	1/14 (7.1%)	Radiocarpal
Pomerance,^21^ 2006	3/17 (17.6%)	Radioscaphoid
Lee et al,^22^ 2023	0/19 (0%)	
Lavernia et al,^23^ 1992	3/21 (14.3%)	Radial Styloid (2), Lunatocapitate (1)
Moran et al,^24^ 2005	4/31 (12.9%)	Midcarpal (4), Radiocarpal (4)
Szabo et al,^25^ 2002	0/22 (0%)	
Wintman et al,^26^ 1995	0/20 (0%)	
Carvalho et al,^27^ 2017	1/14 (7.1%)	SLAC I
Deshmukh et al,^28^ 1999	0/44 (0%)	
van Kampen,^15^ 2024	10/16 (62.5%)	SLAC III (7), SLAC I (2), SLAC II (1)
Luchetti et al,^29^ 2010	0/18 (0%)	
Total	28/265 (10.6%)	

Tenodesis Studies	Arthritis	Pattern

Nienstedt et al,^40^ 2023	2/8 (25%)	SLAC III
Moran et al,^8^ 2006	2/15 (13.3%)	Radiocarpal
Athlani et al,^41^ 2019	4/20 (20%)	STT (2), SLAC (2)
Chabas et al,^42^ 2008	1/19 (5.2%)	SLAC II
Links et al,^43^ 2008	0/21 (0%)	
Neinstedt,^44^ 2013	1/8 (12.5%)	Midcarpal
Pauchard et al,^45^ 2013^47^	4/20 (20%)	Scaphotrapezial (2), SLAC II, SLAC
van Kampen et al,^15^ 2024	6/15 (40%)	SLAC I (3), SLAC III (2), SLAC I (1)
Wagner et al, 2022^46^∗	0/12 (0%)	
Wagner et al, 2022^46^	0/9 (0%)	
Total	20/147 (13.6%)	

Bone-Ligament-Bone Studies	Arthritis	Pattern

Nakamura et al,^12^ 2015	2/13 (15.4%)	SLAC II
Dellarosa et al,^13^ 2016	2/11 (18.2%)	Radiolunate
Marcuzzi and Leigheb,^14^ 2016	2/6 (33.3%)	SLAC I
van Kampen,^15^ 2024	8/12 (66.7%)	SLAC III (4), SLAC I (3), SLAC II (1)
Della Rosa et al,^16^ 2022	0/31 (0%)	
van Kampen et al,^17^ 2015^1^	9/13 (69.2%)	SLAC III (4), SLAC I (2), STT∗
Gray et al,^18^ 2015	11/19 (57.9%)	Midcarpal
Total	34/108 (31.5%)	

Ligamentoplasty Studies	Arthritis	Pattern

Métairie et al,^35^ 2022	8/22 (36.4%)	SLAC III (4), Scaphocapitate (3), SLAC II (1)
Athlani et al,^36^ 2021	0/19 (0%)	
Athlani et al,^36^ 2021∗	0/21 (0%)	
Melone et al,^37^ 2012	3/18 (16.7%)	Periscaphoid
Helfter et al,^38^ 2024	1/21 (4.8%)	SLAC IV
Schweizer and Steiger,^39^ 2002	6/22 (27.3%)	Capitolunate (5), Radioscaphoid (2)
Total	18/123 (14.6%)	

Debridement Studies	Arthritis	Pattern

Kim et al,^30^ 2019	0/42 (0%)	
Fok and Fernandez,^31^ 2015	7/36 (19.4%)	Radial Styloid (6), capitolunate (1)
Darlis et al,^32^ 2006	0/11 (0%)	
Darlis et al,^9^ 2005	0/16 (0%)	
Crespo Romero et al,^33^ 2021	0/20 (0%)	
Ruch and Poehling,^34^ 1996	0/7 (0%)	
Total	7/131 (5.3%)	

∗ Scaphotrapeziotrapezoid (STT).

Further, the tenodesis group had 20 cases of arthritis out of 147 wrists, a 13.6% occurrence. The patterns of arthritis found in the tenodesis group were SLAC, radiocarpal, scaphotrapeziotrapezoid, and midcarpal arthritis. The ligamentoplasty group had 18 cases of arthritis out of 123 wrists, a 14.6% occurrence. The mean occurrence of arthritis among studies was 14.2%. Scapholunate advanced collapse was the most common pattern of arthritis in this group, with scaphocapitate, capitolunate, and periscaphoid arthritis also found among patients.

**Figure 2:**
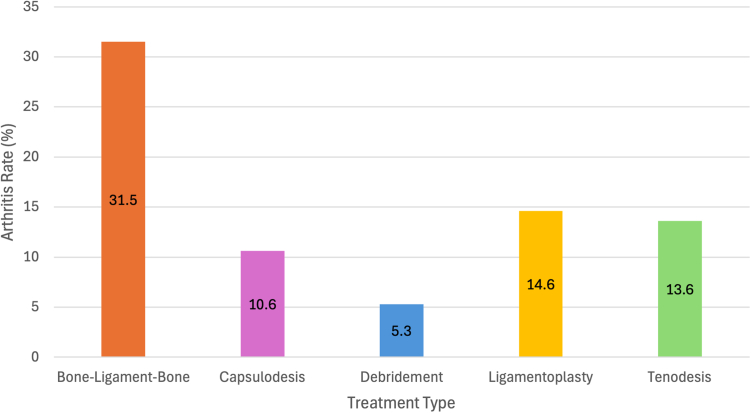
Bar graph displaying the percentage of arthritis development by surgical treatment type at the last follow-up.

Overall, the bone-ligament-bone group had the most occurrences of arthritis, with 34 cases of 108 wrists, a 31.5% rate of occurrence. The predominant patterns of arthritis found were SLAC and midcarpal arthritis, with instances of radiolunate and scaphotrapeziotrapezoid arthritis being less common. Conversely, the debridement group had the fewest occurrences of arthritis, with seven cases of 132 wrists, a 5.3% rate. Only one study resulted in patients with arthritis.^31^ In this study, there were 36 patients, giving a 19.4% occurrence of arthritis. The patterns of arthritis in this study were arthritis at the radial styloid and the capitolunate joint. The rates of arthritis are summarized (Fig. 2).

**Figure 3:**
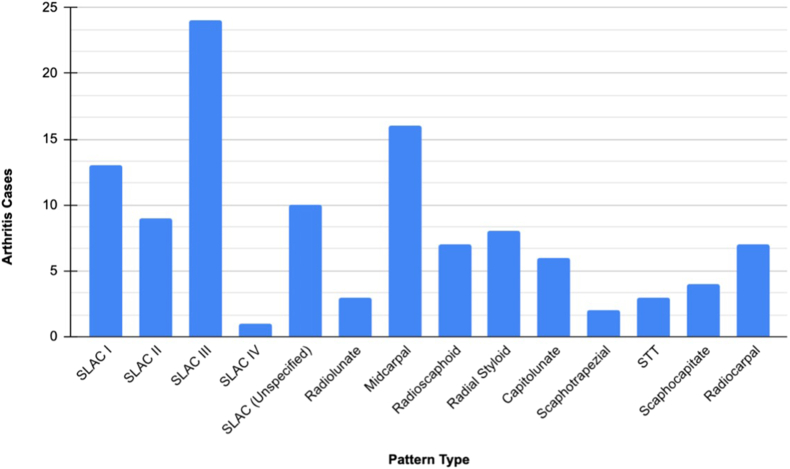
Bar graph displaying the frequency of arthritic patterns developed following SLL injury and repair.

Concerning the specific patterns of arthritis, the most common pattern found among all studies was SLAC III (Fig. 3). These findings underscore the variability in arthritis development following SL ligament repair and highlight the need for further research to refine surgical techniques and patient selection criteria to optimize outcomes.

## Discussion

Although previously published studies examine management and outcomes for SLL injuries, there has yet to be a consensus on which method is best, and none have attempted to find a primary correlation between SLL injury and arthritis development. Consolidating multiple published reports, our results aligned with the findings of Weiss et al,^48^ who first found in a study of 43 wrists using only arthroscopic debridement as management that none had static intercarpal instability pattern changes on follow-up radiographs. Capsulodesis, tenodesis, and ligamentoplasty all had similar rates of arthritis, which means that decisions to use these techniques should be deferred to the individual patient’s presentation and the physician’s preference. Additionally, our results show that arthritis patterns can present in various patterns, with the most arthritic wrists progressing to SLAC III regardless of intervention. Still, according to Watson's findings, we still recommend surgical SL ligament repair for wrists that do not respond to conservative treatment, as untreated injuries will lead to a predictable pattern of arthritis.^6^

Comparing our findings with those of Watson and Ballet,^7^ we agree that the primary arthritic pattern after SLL injury is of the radio scaphoid joint, radial styloid, and capitulate joint—in the study by Watson and Ballet,^7^ SLAC arthritis begins at the radial styloid, moving to the radio scaphoid and capitulate joints. Scaphotrapeziotrapezoid joint arthritis was found in several studies in our review, but was excluded from Watson’s study. Watson mentioned in his findings that the radiolunate joint is unaffected. However, a study in our review, Dellarosa et al,^13^ demonstrated two cases of radiolunate arthritis after SLL injury after bone-ligament-bone surgery.

The demographic analysis of the studies revealed a discrepancy in the gender of patients, with more than a two-to-one ratio of men to women. The mean number of patients per study was acceptable, although some studies included single-digit patient populations. These studies were included to obtain a larger, more representative sample size. There were significantly more patients in the capsulodesis group than in the other treatment types, which limits comparisons. The mean patient age of 39.8 years helped to limit the effect of age-related OA in the results. However, there were still a considerable number of patients >65 years old included in the review, which may impact the quality of the results because of the age-related progression of OA. Although not analyzed in this review, the delay between injury and surgery may play a role in the development of arthritis. No significant literature exists on the development of OA seen in untreated SLL injuries. A documented timeframe of OA development in untreated wrists would clarify the results of our review; one study was found, Pilný et al,^49^ but there was no existence of an English-translated publication.

The final demographic metric, wrist dominance, was relatively evenly represented among studies that reported approximately 60% of injuries to the dominant wrist. However, nearly half of the included studies did not report hand dominance, making it difficult to obtain meaningful information on wrist dominance and arthritis development.

Although our results for SL ligament injury treatment and present patterns of arthritis are precise, our review’s limitations must be considered. First, at least partly, many limitations can be attributed to deficiencies in the literature on arthritic development after an SL ligament injury. Notably, other authors have also discussed the lack of literature.^50^ We were also unable to conduct any solid statistical analysis because of the heterogeneity of the diagnostic methods, surgical techniques, outcome measures, and length of follow-up. Montgomery et al^50^ also showed this statistical omission. Furthermore, the nature of arthritis is a long-term developmental process with a variety of leading secondary factors. Palazzo et al^51^ stated that risk factors of OA can be divided into person-level factors (age, gender, obesity, genetics, and diet) and joint-level factors (injury, malalignment, and abnormal loading of the joints), which interact in a complex manner. Hence, it is difficult to say that SL ligament injuries alone have a direct link to arthritis. This truth emphasizes a greater need for long-term studies in developing arthritis post-SL ligament injuries.

Another limitation of our study is the unique differences between studies. For example, some reports we examined found evidence of arthritis using plain radiographs, whereas others used arthroscopic techniques. When counting the number of arthritis occurrences, we grouped all the patterns of arthritis into one. We only considered the difference between the patterns when we ignored the surgical technique. This decision was because of the difference in diagnosing arthritis between studies. Overall, not having standardization in the discovery of arthritis may demonstrate a lower effect on our research.

Finally, concerning the quality of the studies we examined, the bias among them was relatively low. We used the Downs and Black checklist to assess methodological quality. Only two of the 37 studies investigated scored poorly. The rest were considered fair to excellent, with the vast majority classified as sound sources. However, despite these limitations, the implications demonstrated in this review still have plenty of clinical significance.

Moving forward, more long-term studies on the development of arthritis in the context of SLL injury are needed. Future studies would need to include a randomized controlled trial, as we were unable to find any randomized controlled trial studies that fit within our inclusion criteria. In the future, when conducting these longitudinal studies, it is essential to find ways to distinguish the development of arthritis because of injury or a secondary factor like age, possibly using a trivial cutoff number or grouping patients based on one of these factors. Additionally, our study agrees that there is no consensus on the best management of SLL injuries to prevent arthritis. Still, if conservative management fails, arthroscopic debridement seems to be a leading candidate if indicated for patients. Future studies involving treatment after arthritis has been developed could lead to a broader understanding of SLL tears and wrist pain management.

## Conflicts of Interest

No benefits in any form have been received or will be received related directly to this article.
